# 3D Hierarchical Bi_2_S_3_ Nanostructures by Polyvinylpyrrolidone (PVP) and Chloride Ion-Assisted Synthesis and Their Photodetecting Properties

**DOI:** 10.1186/s11671-015-0993-1

**Published:** 2015-07-09

**Authors:** Taotao Ding, Jiangnan Dai, Juan Xu, Jin Wang, Wu Tian, Kaifu Huo, Yanyan Fang, Changqing Chen

**Affiliations:** Wuhan National Laboratory for Optoelectronics, Huazhong University of Science and Technology, 1037 Luoyu Road, Wuhan, 430074 Hubei People’s Republic of China

**Keywords:** Bi_2_S_3_, 3D, Photoresponse, Morphology, Surfactants, KCl

## Abstract

A solvothermal method has been employed to synthesize bismuth sulfide (Bi_2_S_3_) with three-dimensional (3D) hierarchical architectures. The influences of different types of surfactants and Cl^−^ species on the size and morphology were investigated. A possible formation mechanism was also proposed on the basis of time-dependent experiments. The photoresponse properties show that the conductivity of Bi_2_S_3_ micro-flowers is significantly enhanced and the photocurrent is approximately two orders of magnitude larger than the dark current. The response and decay times are estimated to be 142 and 151 ms, respectively. It is expected that hierarchical architectures Bi_2_S_3_ may provide a new pathway to develop advanced nanomaterial for high-speed and high-sensitivity photoelectrical switches and photodetecting devices.

## Background

Bismuth sulfide (Bi_2_S_3_) that is an important member of group V–VI binary semiconductors, has drawn increasing attention in solar cells [1], photodetectors [2–10], gas sensors [11], Schottky diode [12], lithium-ion battery [13], X-ray computed tomography imaging (CT) [14], and thermoelectric devices [15]. In recent years, various morphologies of Bi_2_S_3_ micro-/nanostructures, including one-dimensional (1D) nanoribbons/nanowires [16–19] and nanorods [20], two-dimensional (2D) nanosheets [10], and three-dimensional (3D) hierarchically complex architectures [13, 16], have been fabricated. Among them, 3D hierarchically porous and hollow nanostructures have showed enhanced properties for applications in lithium-ion batteries [21], photocatalysts [22], and gas sensors [23], because of their large surface area and facile electron (ion) transport. 3D hierarchical Bi_2_S_3_ nanoarchitectures are usually produced through solution-based synthesis [4, 13, 24, 25], and chemical vapor deposition routes [26] and their excellent physical and chemical properties have been revealed. In 2009, Li et al. built their photodetectors with Bi_2_S_3_ core-shell mircospheres. The light current increased by 1.1 times upon exposure to the simulated sunlight. The signal-to-noise ratio (SNR) of the photodetectors was probably too low [10]. The devices based on the Bi_2_S_3_ hierarchical architectures reported by Xiao et al. had their response time and decay time of 0.5 and 0.8 s, respectively [2]. That cannot meet the demand of high-speed photodetectors. Besides, the methods they employed were too complex, compared with hydrothermal or solvothermal approaches. At the same year, Li et al. reported a photodetector based on the Bi_2_S_3_ hierarchical architectures, with a fast response time of ~50 ms via a hydrothermal method [3]. However, their light current was about 30 nA with illumination of 100 mW cm^2^ (AM 1.5), which may be too low for high-performance photodetectors. Therefore, fast and high response photodetectors based on Bi_2_S_3_ are still a challenge for practical applications. To improve the crystal quality and morphology of Bi_2_S_3_, nanostructures may be a good route to enhance their photodetecting properties. It has been reported that chloride ion (Cl^−^) could monitor the crystal growth of Cu_2_O [27], silver nanocubs [28], and silver nanoparticles [29], because Cl^−^ could retard the nucleation and growth and reduce the surface energy by binding strongly to seeds. Thus it is reasonable to conjecture that the Cl^−^ could affect the crystallinity and growth of 3D hierarchical Bi_2_S_3_ considering the similar crystalline structure and growth behavior of Bi_2_S_3_ and Cu_2_O. However, there is no report to prepare Bi_2_S_3_ nanostructures by introducing Cl^−^ to monitor the morphology of Bi_2_S_3_ during solvothermal process. Moreover, the influence of surfactants on the morphologies of 3D hierarchical Bi_2_S_3_ has not been investigated systematically. For example, Jiang et al. reported that they had synthesized flower-like Bi_2_S_3_ by an ionic liquid-assisted templating route [30]. In 2010, the flower-like Bi_2_S_3_ had been synthesized via a hydrothermal method by Tang et al [31]. And the similar Bi_2_S_3_ had also been obtained by Wang et al. via the same hydrothermal method and assembled into the dye-sensitized solar cells with a good performance [32]. Chen et al. reported that they had synthesized ultrathin Bi_2_S_3_ nanosheets via an organometallic synthetic route [10].

In this paper, we report a facile synthetic route to synthesize 3D hierarchical flower-like Bi_2_S_3_ consisted of nanowires via a solvothermal approach. Three types of surfactants including polyvinylpyrrolidone (PVP), sodium dodecyl sulfate (SDS), and centrimonium bromide (CTAB) were employed in the synthesis of Bi_2_S_3_ nanostructures, and PVP shows more manifest effects on the morphologies than the other two surfactants. The potassium chloride was added first in the solution to investigate the influence of chloride ions on 3D hierarchical Bi_2_S_3_ during solvothermal process. Our results demonstrate that Cl^−^ plays a critical role on monitoring the shapes of Bi_2_S_3_ nanostructures. A possible formation mechanism of 3D Bi_2_S_3_ hierarchical nanostructures is proposed. Furthermore, a photodetector has been constructed based on as-prepared 3D Bi_2_S_3_ hierarchical nanostructures. The results show that the photocurrent is enhanced by two orders of magnitude compared with the dark current and the response time and decay time are estimated to be 142 and 151 ms, respectively, indicating promising applications of the as-prepared 3D hierarchical Bi_2_S_3_ for photodetecting and photoelectric switches.

## Methods

### Materials Synthesis

Bi(NO_3_)•5H_2_O, thiourea (TU), polyvinylpyrrolidone (PVP), and ethylene glycol (EG) were purchased from Sinopharm Chemical Reagent Co., Ltd., (Shanghai) without further purification.

In a typical procedure, 0.6 g Bi(NO_3_)•5H_2_O, 0.3 g TU, and 0.1 g PVP were added successively into 40 mL EG. The resulting mixture was sonicated to obtain a clear, yellow solution, which was then transferred into a 100-mL Teflon-lined autoclave and heated at 60 °C for 24 h. Finally, the sample was collected and washed with distilled water and ethanol for three times, and then dried at 60 °C for 12 h in a vacuum oven. This final sample was designated as V-Bi_2_S_3_.

To investigate the influence of surfactants on the morphologies of the Bi_2_S_3_ micro-structures, another two surfactants sodium dodecyl sulfate (SDS) and centrimonium bromide (CTAB) were selected. Moreover, potassium chloride with different amounts was added to investigate the effects of chloride ions on the final morphologies. Each control experiment was performed in the same conditions except the change of the surfactants and chloride concentrations.

The morphologies, structures, and compositions were characterized by field emission scanning electron microscopy (FE-SEM, FEI Nova NanoSEM 450) and transmission electron microscopy (TEM; FEI Tecnai G20). X-ray powder diffraction (XRD) characterization was performed on Shimadzu XRD-7000s diffractometer equipped with Cu Kα radiation (*λ* = 0.15418 nm). X-ray photoelectron spectra (XPS) were characterized with Kratos AXIS Ultra DLD-600W X-ray photo electron spectroscopy.

### Device Fabrication

The photodetectors were fabricated by a simple drop-casting method. Typically, 10 mg V-Bi_2_S_3_ was first suspended in 2 mL ethanol by sonication. The Au interdigital electrodes (1.5*1.0 cm, the electrode gap size is 1 μm) on Al_2_O_3_ substrates were cleaned by distilled water, ethanol, and acetone successively for 15 min, respectively. And then 10 μL of suspension was dropped on the Au electrodes. Finally, the devices were put in an oven at 30 °C for 12 h. Electrical property measurements and photo-sensing tests were conducted in ambient condition by a semiconductor characterization system (Keithley 2420) and a solar simulator (Newport 91160-1000) in the dark and under simulated AM 1 and 1.5 illumination.

## Results and Discussion

### Crystal Structure

**Fig. 1:**
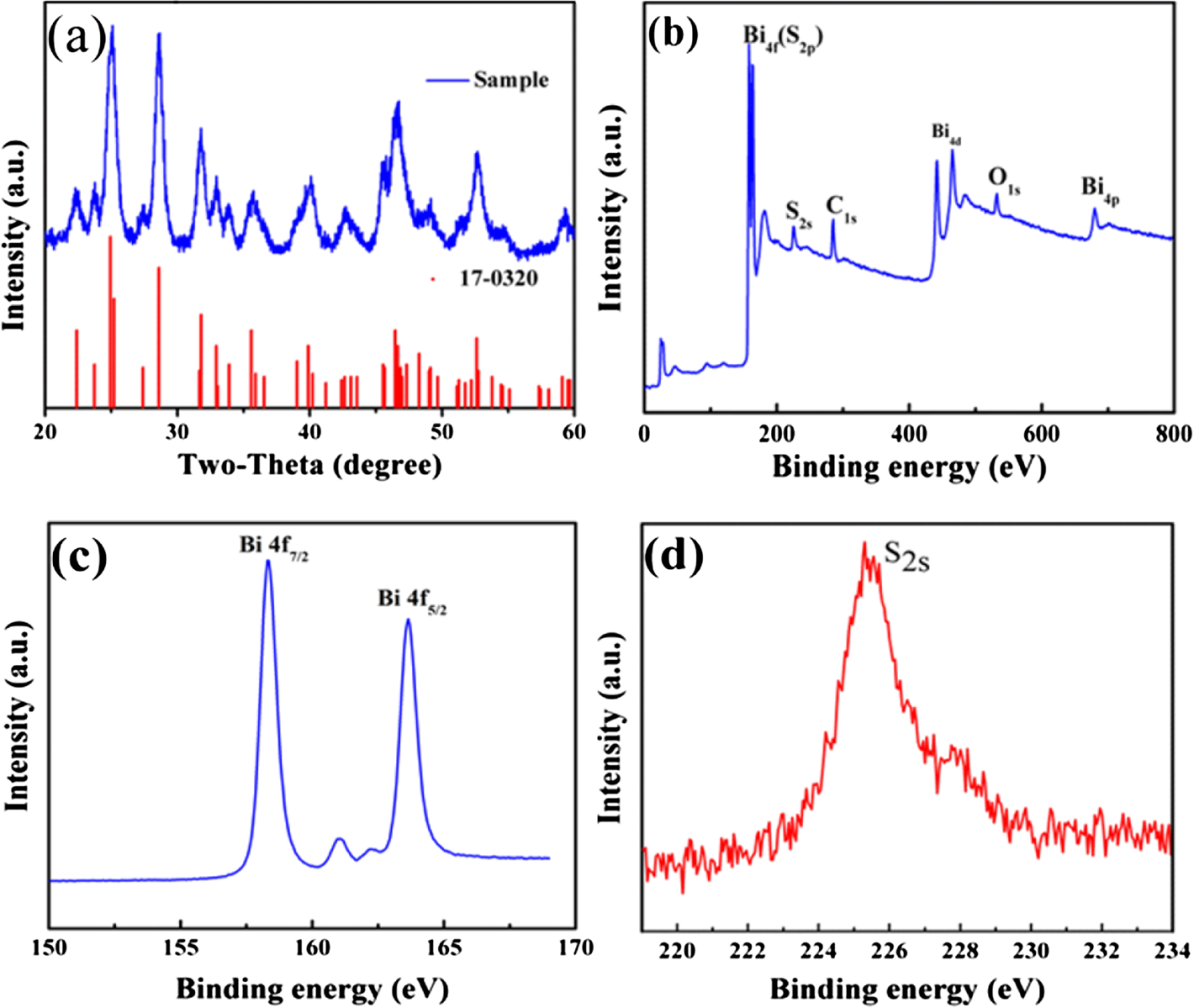
**a** The XRD pattern of the V-Bi_2_S_3_. The pattern shown at the *bottom* is the standard XRD card of Bi_2_S_3_ (JPCDS no. 17-0320). **b**–**d** The full spectra, Bi2f, and S2s region of the XPS spectrum of V-Bi_2_S_3_, respectively

Figure 1a is the XRD pattern of the product obtained via a one-pot solvothermal method of using bismuth nitrate as the precursor and ethylene glycol as the solvent. All the peaks can be indexed to the orthorhombic Bi_2_S_3_ phase (JCPDS no. 17-0320), and no characteristic peaks of any other phases and impurities are observed. In another investigation, the average crystalline size was derived from Scherrer formula as shown below:

$$ D=\frac{k\lambda }{B \cos \theta } $$

where *D* is the average crystalline size, *k* is a constant whose value is typically 0.9 of non-spherical crystals, *B* is the full width at half maximum (FWHM) of the diffraction peak (in radians) that has the maximum intensity in the diffraction pattern, *λ* is the wavelength of incident X-ray beam (0.154184 nm), and *θ* is diffraction angle or Bragg angle. From this formula, the average crystalline size of Bi_2_S_3_ was calculated 12.27 nm. The full spectrum of XPS shows four distinct peaks corresponding to bismuth, carbon, sulfur, and oxygen, respectively (Fig. 1b). The peaks for O can be attributed to the absorbed oxygen species on the sample surface, which is commonly observed for samples exposed to the atmosphere and more pronounced for ultrafine powders with high surface areas. The C is from the absorbed carbon species during XPS measurement. The fine-spectrum of Bi is shown in Fig. 1b, two peaks located at ca. 163.65 and 158.3 eV (Fig. 1b) are assigned to the Bi 4f_5/2_ and Bi 4f_7/2_, respectively. Two peaks between the Bi4f7/2 and Bi 4f5/2 can be ascribed to the S 2p3/2 and S 2p1/2 that located 160.95 and 162.4 eV [33, 34]. The binding energy of located at 225.3 eV can be attributed to the S^2−^ (2s) Fig. 1c. The reason for the asymmetric S2s peak is that there is a combination of both S_8_ which is expected at 228 eV, and SO_x_ species [35], where *x* < 3 (that differs from metal sulfite salts), are typically at ~230 eV. Metal sulfites are typically found at ~230 eV. S_8_ is a byproduct of the reaction that is difficult to remove during purification.

### The Morphology and Proposed Formation Mechanism

**Fig. 2:**
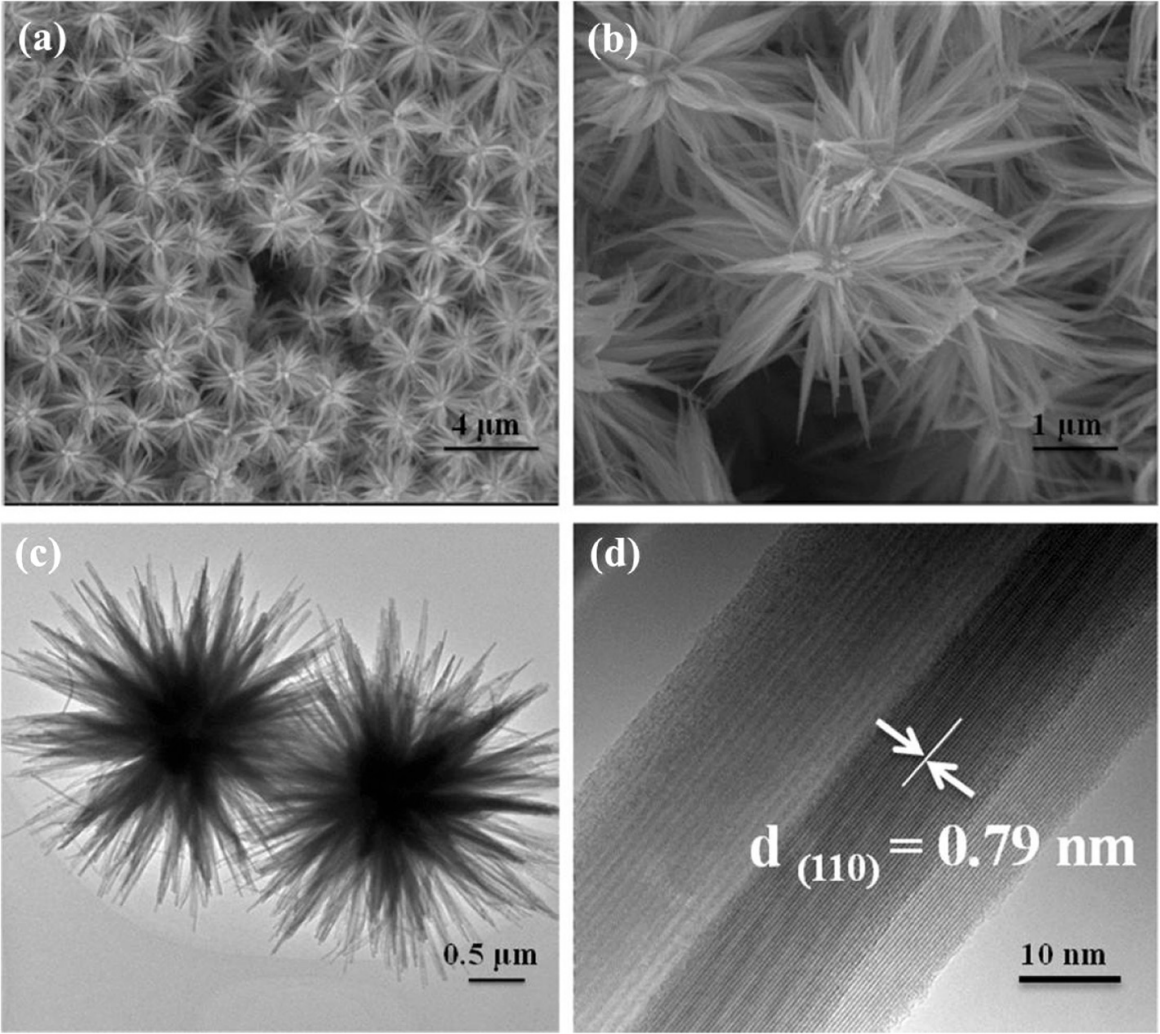
**a**, **b** Typical SEM images of V-Bi_2_S_3_. **c**, **d** TEM image and HRTEM image of V-Bi_2_S_3_, respectively

Figure 2a, b are low- and high-magnification FE-SEM images, revealing flower-like Bi_2_S_3_ nanostructures are produced. The flower-like Bi_2_S_3_ nanostructures are composed of numerous nanowires with diameters of about 12 nm and length up to 1 μm. TEM images further reveal that the petals burst forth. The HRTEM image (Fig. 2d) reveals the lattice fringe of a nanowire with a regular spacing of 0.79 nm, corresponding to the (110) plane of orthorhombic Bi_2_S_3_.

**Fig. 3:**
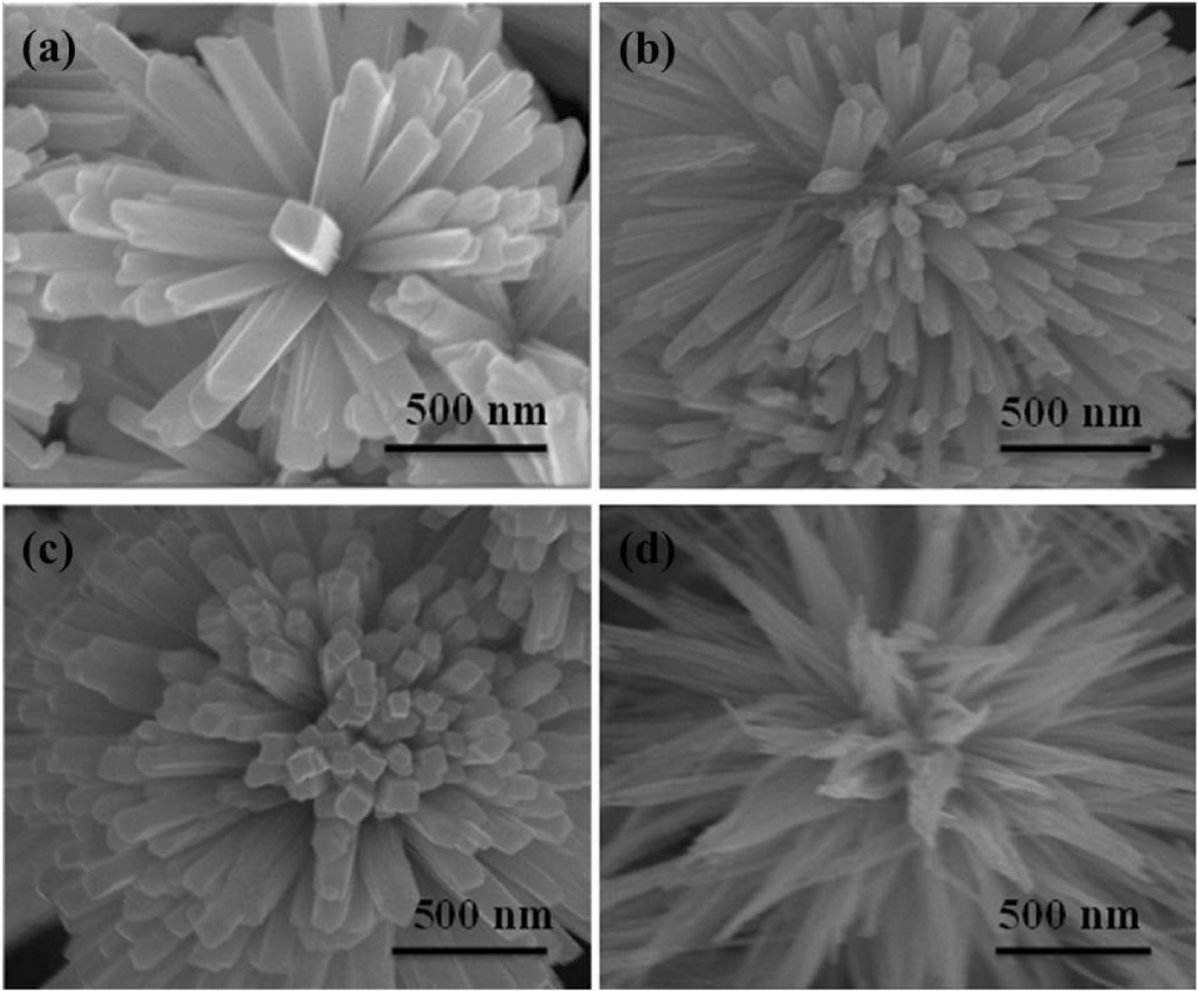
The typical SEM images of Bi_2_S_3_ structures synthesized **a** blank, without surfactants, **b** CTAB, **c** SDS, and **d** PVP

It had been reported that surfactants have a very important influence on the morphologies of the products via solvothermal reaction [36–38]. Herein, three different types of surfactants, i.e., SDS, CTAB, and PVP, were added into the solvent-thermal proceeds to investigate the surfactant-dependent morphologies of Bi_2_S_3_. Without adding any surfactant, Bi_2_S_3_ micro-flowers consisted of nano-cuboids with 110 nm in diameter and 0.5 μm in length could be fabricated, as is shown in Fig. 3a. When SDS and CTAB were added in the solution, the overall morphologies of Bi_2_S_3_ have no obvious change. However, when PVP is added in the solution, flower-like Bi_2_S_3_ consisting of nanowires could be produced. The aforementioned results indicated that the PVP will be beneficial for the growth of thin nanowires compared with SDS and CTAB surfactants. The main reason may stem from the chelating between the oxygen (and/or nitrogen) of pyrrolidone from the PVP molecules and Bi^3+^, resulting in selective absorption and growth of various crystallographic planes of Bi_2_S_3_ [38]. In addition, PVP may also play a key role in inducing the formation of the flower-like Bi_2_S_3_ consisting of nanowires [39]. PVP molecules absorbed on the surface of Bi_2_S_3_ nanoparticles could reduce total surface energy of the reaction system; as a consequence, flower-like Bi_2_S_3_ assembled by nanowires is produced.

**Fig. 4:**
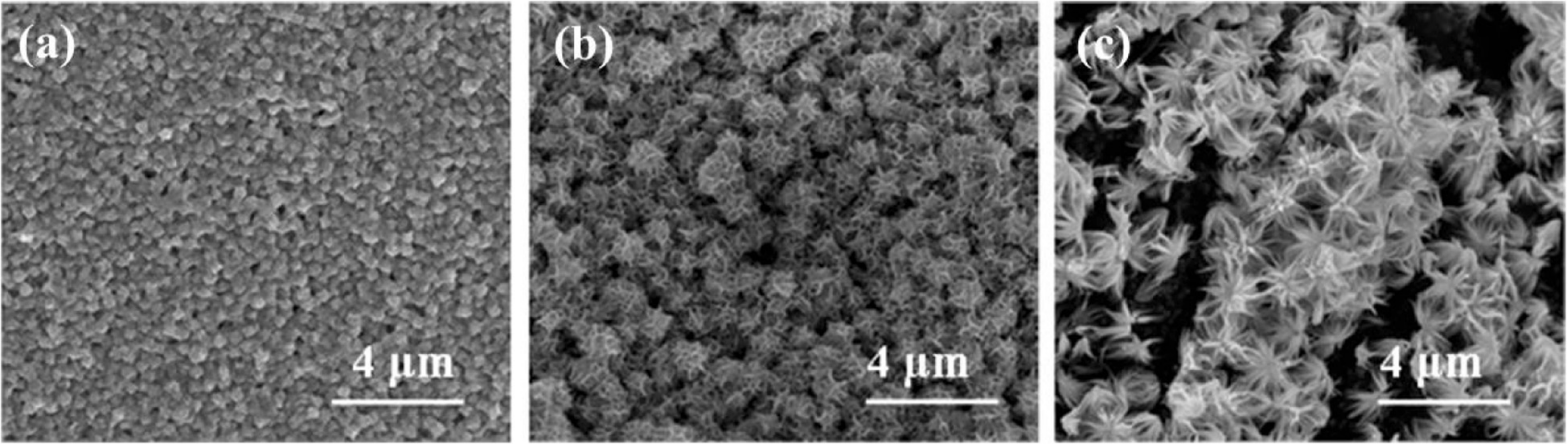
FE-SEM images of the products obtained at different time periods: **a** 2 h, **b** 6 h, and **c** 24 h

To further reveal the formation process of the 3D flower-like Bi_2_S_3_ nanostructures, a series of time-dependent experiments was performed. Figure 4 shows the evolution of morphology at 60 °C elucidated after different reaction periods. If the reaction was carried out for 2 h, Bi_2_S_3_ microspheres with a diameter of 500 nm were produced (Fig. 4a). As the reaction time was prolonged to 6 h (Fig. 4b), microspheres became lager and carved by many folds. When the reaction time was further extended to 24 h, the micro-flowers consisting of nanowires were finally obtained (Fig. 4c).

The bismuth–thiourea system has been well-developed to prepare bismuth sulfide in various forms [40, 41]. It was observed that the yellow color in solution faded indicating Bi^3+^-Tu complexes decomposed to form a mass of Bi_2_S_3_ nuclei. Spherical cores appeared at the initial stage. The oxygen (and/or nitrogen) atoms of pyrrolidone units of PVP chemically interact with the newly formed nuclei, thus the nuclei were stabilized.

$$ B{i}^{3+}+nTu\rightleftharpoons {\left[ Bi{(Tu)}_n\right]}^{3+} $$

$$ N{H}_2CSN{H}_2\rightleftharpoons C{H}_2{N}_2+{H}_2S $$

$$ 2B{i}^{3+}+3{H}_2S\to B{i}_2{S}_3+6{H}^{+} $$

$$ B{i}_2{S}_3+ nPVP\to B{i}_2{S}_3{(PVP)}_n $$

**Fig. 5:**
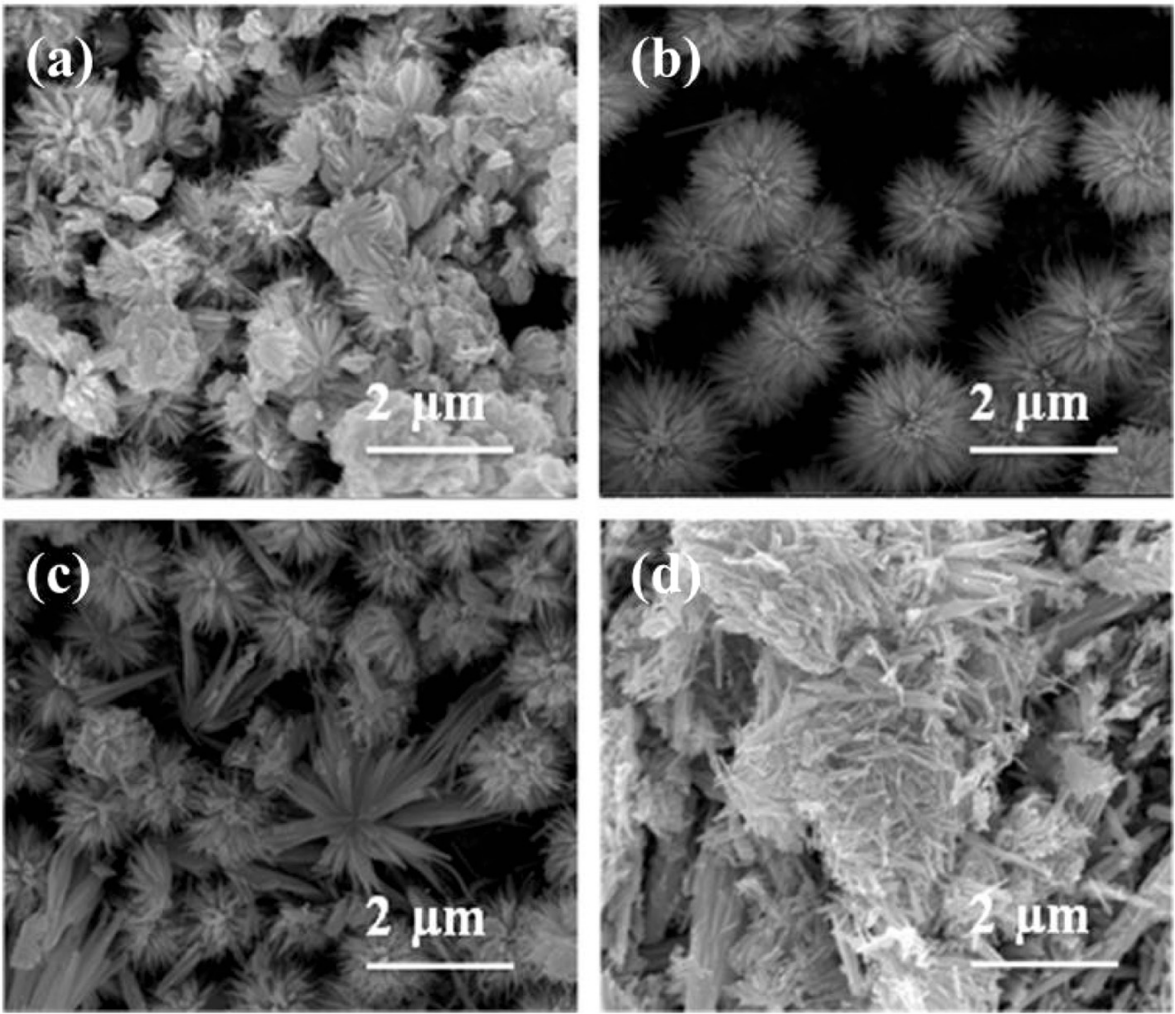
FE-SEM images of the samples synthesized with different quantity of KCl: **a** 0.01 g, **b** 0.03 g, **c** 0.05 g, and **d** 0.1 g

Besides, it is found that the Cl^−^ has an important effect on the final morphologies of final Bi_2_S_3_. When the KCl was added, the flower-like Bi_2_S_3_ had changed their morphologies immediately, as suggested by Fig. 5. If the low amount of KCl (0.01 g) was added to system, flower-like Bi_2_S_3_ began to assemble into a sphere-like structure. More KCl (Fig. 5b) was added, and Bi_2_S_3_ microspheres consisted of quantities of individual nanowires were obtained. The wire-like petals had a diameter and length of 12 nm and 1 μm, respectively. However, too much KCl (Fig. 5c, d) would lead to an opposite outcome in that some sphere-like structures transformed to flower-like again with petals turning belt-like. The possible reason is that chloride ions can reduce the surface energy of some facets of the seeds by binding strongly to them, probably by the way of coordination, thus resulting in the formation of microspheres of Bi_2_S_3_. In addition, these Cl^−^ species can prevent the microspheres from aggregating by providing electrostatic repulsion between the microspheres [28, 29].

### The Photoresponse Properties of the V-Bi_2_S_3_

**Fig. 6:**
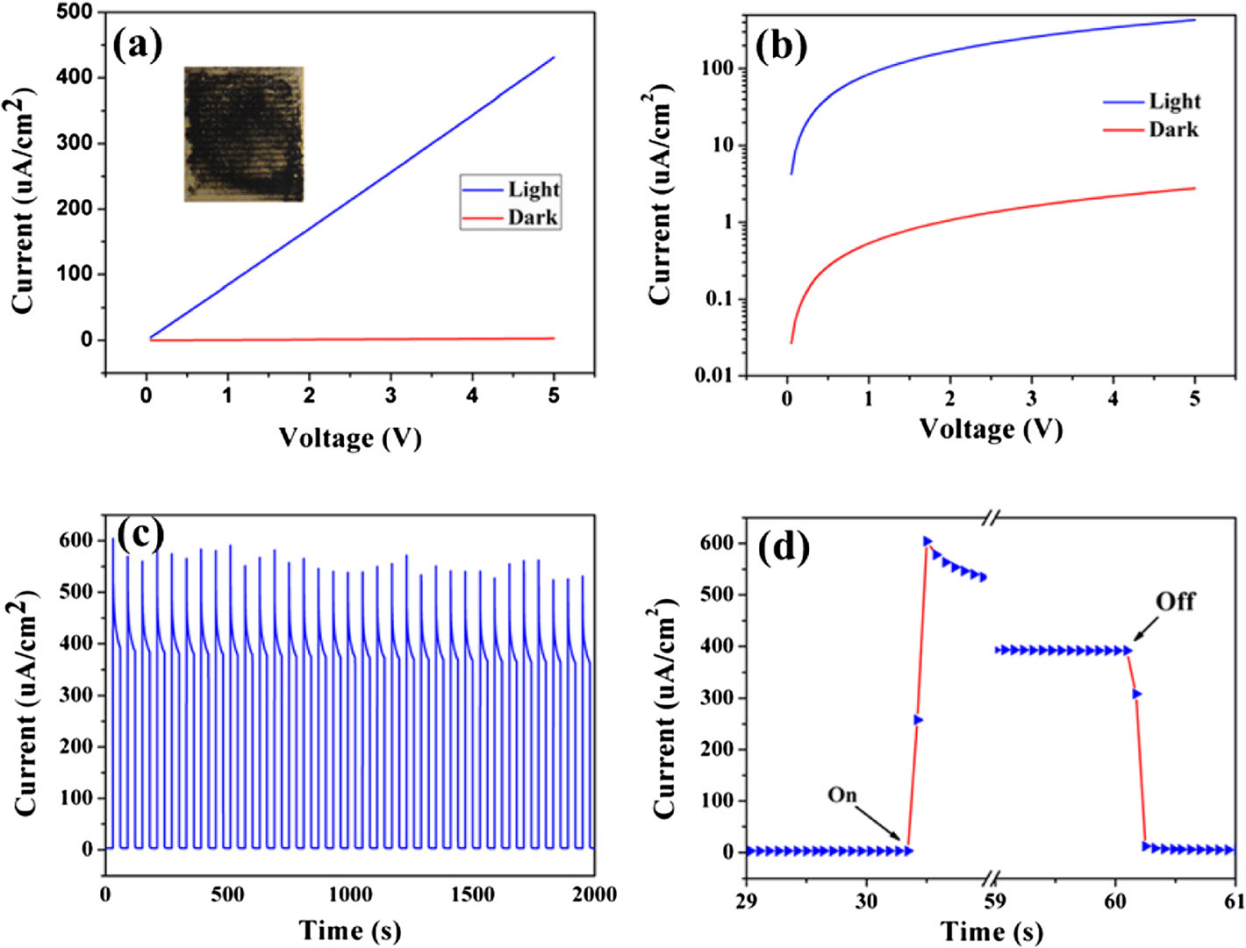
Photoresponsive sensitivity of the V-Bi_2_S_3_ architectures as a representative system was studied. **a** The *I-V* characteristic of a device in the dark and under simulated A M 1.5 illumination. **b** Logarithmic plot of (**a**). **c** Time dependence of current of Bi_2_S_3_ micro-flower at a bias of 5 V in the dark and under simulated A M 1 illumination. **d** The enlarged portion of the 29–59 s and 59–61 s

Photodetectors and optical switches are indispensable elements in memory storage and optoelectronic circuits in imaging techniques and light-wave communications [42]. Unfortunately, conventional photodetectors are usually in film or bulk configurations with higher power consumption compared with the photodetectors constructed by micro- or nanoscale materials. We choose V-Bi_2_S_3_ as a representative micro- and nanomaterial to evaluate the potential application in photodetectors. The schematic illustration of Bi_2_S_3_ micro-flower-based photodetector is shown in the inset of Fig. 6a. Evidently, the linear behavior of the *I-V* curve (Fig. 6a) for both dark current and photocurrent with a bias from 0 to 5 V indicates a good ohmic contact. Under light illumination, a distinct photo-excited current was observed, demonstrating these micro-flower Bi_2_S_3_ have an excellent photovoltaic response upon light illumination. The photocurrent increases with bias voltage increases, showing that the separation of photo-generated electrons and holes is more efficient at a high bias voltage. In addition, the photocurrent significantly increases by ca. two orders of magnitude compared with the dark state (Fig. 6b) which indicates high photosensitivity. High photoresponse should result from the increased charge carrier concentration via direct electron-hole pair creation under light illumination and enhanced the conductivity of Bi_2_S_3_; Fig. 6c depicts the photoresponse as a function of time with the light regularly chopped at a bias of 5 V to reveal the stability and response capability. The photocurrent quickly reaches to a maximum value (the steady state), and then rapidly returned to its initial ones (the normal state) once the light was turned off, revealing the Bi_2_S_3_ micro-flowers respond quickly to the light. Such on–off cycles were repeated several times, and no detectable degradation was found, showing its excellent stability and reproducible behavior. It is generally defined that the response time as the time needed to recover to 10 % of the maximum photocurrent equal to the calculated response and recovery time for our photodetector is calculated to be 142 and 151 ms at a bias of 5 V. The above results indicate that the photodetector based on Bi_2_S_3_ micro-flowers has a good stability and responds quickly to light, suggesting promising applications of Bi_2_S_3_ micro-flower in photodetector and photoelectrical switches. More importantly, the Bi_2_S_3_ micro-flowers as well as the relevant photosensitive devices presented in this paper were very easy and do not need complex equipment and procedures, thus offering probability for low-cost and large-scale circuit integration.

## Conclusions

In summary, a facile solvothermal procedure has been developed for large-scale production of 3D micro-structures consisted of ultra-long Bi_2_S_3_ nanowires with a diameter of 12 nm and axial dimension of up to 1 μm. The influences of surfactant, KCl, and time on the final morphologies of Bi_2_S_3_ nanostructures have been investigated, and the growth mechanism is proposed. The capping effect of the PVP and chloride ions and the specific amount of Cl^−^ species seem to be the most pivotal factors in guiding the formation of Bi_2_S_3_ micro-flowers and microspheres. A high efficient photodetector was constructed based on Bi_2_S_3_ micro-flowers. The photoresponse properties show that the conductivity of Bi_2_S_3_ micro-flowers is significantly enhanced and the photocurrent is approximately two orders of magnitude larger than the dark current. The response and decay times are estimated to be 142 and 151 ms, respectively, suggesting promising applications in photodetectors.
